# Changes in Malaria Patterns in Comoros from 2010 to 2021: A Comparative Study with Sub-Saharan Africa

**DOI:** 10.3390/tropicalmed10050138

**Published:** 2025-05-19

**Authors:** Sheng Zhou, Linxin Yu, Jianming Liang, Wei Xie, Guoming Li, Changsheng Deng, Jianping Song, Guanyang Zou, Yinhuan Chen

**Affiliations:** 1 Artemisinin Research Center, Guangzhou University of Chinese Medicine, Guangzhou 501405, China; shengzhougzm@163.com (S.Z.); ylx15180549955@126.com (L.Y.); liangjianming@gzucm.edu.cn (J.L.); liguoming2015@sina.cn (G.L.); dcs@gzucm.edu.cn (C.D.); songjp@gzucm.edu.cn (J.S.); 2 School of Public Health and Management, Guangzhou University of Chinese Medicine, Guangzhou 510006, China; 3 Institute of Science and Technology Park, Guangzhou University of Chinese Medicine, Guangzhou 510540, China; glen-tse@hotmail.com; 4 The First School of Clinical Medicine, Guangzhou University of Chinese Medicine, Guangzhou 501405, China

**Keywords:** malaria, Comoros, sub-Saharan Africa, trend analysis, estimated annual percent change (EAPC), decomposition analysis (DA), risk factors, COVID-19

## Abstract

Background: Recent setbacks in malaria control in Comoros demand a reassessment of its evolving epidemiology. Methods: Using the Global Burden of Disease (GBD) Study 2021 data, we analyzed malaria trends from 2010 to 2021, stratified by sex. We quantified the contributions of demographic and epidemiological factors to these trends and identified risk factors for malaria-related disability-adjusted life years (DALYs). Results: From 2010 to 2021, malaria cases, deaths, and DALYs in Comoros fell by −90.22%, −94.44%, and −94.88%; and the corresponding age-standardized rates declined with EAPCs of −18.70% (95% CI: −33.77 to −0.20), −23.89% (95% CI: −36.58 to −8.66), and −24.49% (95% CI: −36.88 to −9.66), with steeper declines in males. Nevertheless, all indicators increased in 2018 and again in 2021. In sub-Saharan Africa, only cases increased, while other metrics declined slightly. In Comoros, incidence shifted mainly to adults ≥25 years, unlike sub-Saharan Africa, where children < 5 years were most affected. Population growth drove increases in cases, deaths and DALYs, whereas epidemiological shifts had the opposite effect. Child underweight was the leading risk factor for malaria DALYs. Conclusions: Existing interventions can achieve malaria control in Comoros; however, rebounds in 2018 and 2021 highlight the need to identify and address drivers of resurgence.

## 1. Introduction

Malaria is a life-threatening disease caused by protozoan parasites of the genus *Plasmodium*, which invade and lyse human erythrocytes, leading to severe systemic complications. Transmission occurs predominantly through the bite of an infected female *Anopheles* mosquito. Five species—*P. falciparum*, *P. vivax*, *P. ovale*, *P. malaria*, and *P. knowlesi*—infect humans, with *P. falciparum* responsible for the majority of severe and fatal cases [1]. Clinically, malaria can result in severe anemia, cerebral malaria, renal failure, splenomegaly, B-cell malignancies, intra-uterine growth restriction, and pre-term delivery [2]. In 2023, malaria remained a major global health threat, with an estimated 263 million cases and 597,000 deaths worldwide. The WHO African Region bore the heaviest burden, accounting for 94% of cases and 95% of deaths, disproportionately affecting children under 5 [3]. Beyond its health impact, malaria also constrains economic development: in high-transmission settings, national gross domestic product (GDP) per capita declines by approximately 1.3% [4]. By the end of 2019, joint global efforts had averted an estimated 1.5 billion cases and 7.6 million deaths. During this time, significant economic growth, improvements in infrastructure and housing, rapid urbanization, and overall advancements in health systems and population health occurred globally [5]. However, the emergence of COVID-19 in late 2019 reversed many gains, triggering a marked resurgence of malaria, especially in the WHO African Region [3].

Comoros, an archipelago between the east African coast and northern Madagascar, is among the countries most severely affected by malaria. The Union of the Comoros comprises three volcanic islands, namely, Grande Comore (Ngazidja), Mohéli (Mwali), and Anjouan (Ndzuwani), while a fourth island, Mayotte (Maoré), remains under French jurisdiction [6]. With a population of 850,387 [3] and widespread poverty [7], Comoros experiences year-round malaria transmission that peaks during the November–April rainy season [6]. In 2023, the country reported 63% more cases and deaths than in 2015 [3], indicating a divergence from the GTS trajectory [8] and underscoring the need to prioritize the national malaria program. At present, few studies have examined the changes in malaria patterns in Comoros over the past decade or explained the drivers underlying the recent resurgence. In this study, leveraging the Global Burden of Disease 2021 (GBD) study database, we analyzed malaria control trends in Comoros from 2010 to 2021, as well as the factors driving those trends, stratified by sex. We also examined the distribution of the malaria burden in four specific years (2010, 2015, 2020, and 2021) across different population and age groups and identified risk factors for malaria-related DALYs. Finally, we compared the findings for Comoros with those for sub-Saharan Africa to analyze the causes of malaria resurgence and to provide an evidence base for developing malaria control measures tailored specifically to Comoros.

## 2. Method and Materials

### 2.1. Data Sources

The GBD 2021 database, developed by the Institute for Health Metrics and Evaluation (IHME), systematically records epidemiological and disease burden indicators for 371 diseases and injuries across different populations, age groups, regions, and years. Through DisMod-MR 2.1, spatiotemporal Gaussian process regression (ST-GPR), the Cause-of-Death Ensemble model (CODEm), and customized statistical models, the GBD generates comparable estimates of epidemiological and burden metrics [9]. Disease burden metrics include years of life lost (YLLs), years lived with disability (YLDs), and disability-adjusted life years (DALYs). The 95% uncertainty interval (UI) of estimates is calculated by taking the 2.5th and 97.5th percentiles from an ordered set of 500 draws [9]. In this study, we extracted data on malaria, including the number of malaria cases, deaths, DALYs, and their crude and age-standardized rates (per 100,000), from 2010 to 2021 using the GBD Results Tool (https://vizhub.healthdata.org/gbd-results/; accessed on 16 May 2024), and stratified the data by sex, age, period, and region. Detailed information on these indicators provided by GBD enabled an accurate assessment of the malaria epidemiological status, disease burden, and temporal trends of these metrics. All analytical procedures followed the Guidelines for Accurate and Transparent Health Estimates Reporting (GATHER) [10].

### 2.2. Disease Definition

Malaria is a potentially fatal disease caused by *Plasmodium* parasites that infect human red blood cells and are primarily transmitted by infected female *Anopheles* mosquitoes. Five species can infect humans, namely, *P. falciparum*, *P. vivax*, *P. malaria*, *P. ovale*, and *P. knowlesi*, with *P. falciparum* being the most prevalent and lethal [1]. According to the International Classification of Diseases 11th Revision (ICD-11), malaria is classified under the following codes: 1F42, 1F45, 1F44, 1F40, 1F43, 1F41, and KA64Y (https://icd.who.int/browse/2024-01/mms/en; accessed on 1 January 2022).

### 2.3. Disability-Adjusted Life Year (DALY)

The DALY is calculated by summing years of life lost (YLLs) and years lived with disability (YLDs), and it has been widely employed in evaluating the malaria burden [11]. It can quantify the overall health loss due to malaria since YLLs represent premature mortality from malaria, while YLDs stand for the deterioration in health caused by malaria.

### 2.4. Risk Factors

Methods for estimating GBD risk factors within a comparative risk assessment framework have been detailed in previous studies [12]. In this GBD 2021 iteration, 88 risk factors were included. Only two such factors, child underweight and child stunting, had non-zero contributions to malaria deaths and DALYs.

### 2.5. Relative Change and Estimated Annual Percentage Change

Relative change (RC) refers to the proportion of change in an observed value relative to its baseline value over a specified period. RC = (Value _final_ − Value _baseline_)/Value _baseline_ × 100% [13]. RC > 0 indicates an increase relative to the baseline; RC < 0 indicates a decrease relative to the baseline; and RC = 0 indicates no change relative to the baseline. The estimated annual percent change (EAPC) has been extensively used in previous studies to depict trends in the age-standardized rates (ASRs) of incidence, mortality, and DALYs [14]. The steps for calculating EAPC are as follows: (1) take the natural logarithm of ASR (y = ln (ASR)); (2) establish a linear regression model with the year as the independent variable (y = α + βx + ε), where x represents the year, y is the natural logarithm of ASR, α is the intercept, β is the slope, and ε is the random error; and (3) calculate EAPC based on the slope of the regression line (EAPC = 100 × (e^β^ − 1)). The 95% confidence intervals (CIs) for EAPC are obtained from this fitted model. When both the EAPC and its 95% confidence interval (CI) exceed zero, the trend is increasing; when both are below zero, the trend is decreasing; and if the CI includes zero, the trend is not statistically significant [14]. In this study, RC and EAPC were employed to evaluate the shifting trends in the number of malaria cases and deaths, as well as DALYs and their ASRs for malaria.

### 2.6. Decomposition Analysis

Decomposition analysis, initially proposed by Das Gupta et al., can assess the underlying factors driving changes in epidemiological and disease burden indicators [15]. We applied this method to determine whether the observed rise or decline in malaria-related indicators was mainly attributable to demographic changes, such as population growth and ageing, or to epidemiological shifts.

### 2.7. Statistical Analysis and Visualization

All statistical analyses and visualizations were undertaken using R software (version 4.4.1).

## 3. Results

### 3.1. Trend Analysis

**Figure 1:**
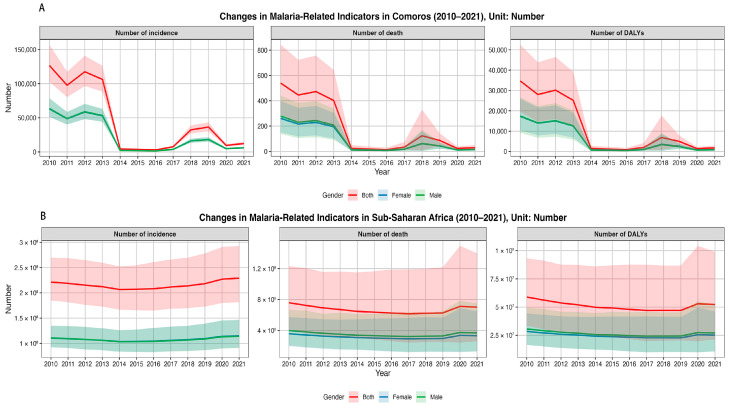
Temporal trends in the number of malaria cases, deaths, and DALYs in Comoros and sub-Saharan Africa from 2010 to 2021, stratified by total population, males, and females: (**A**) Comoros; (**B**) sub-Saharan Africa. DALYs, disability-adjusted life years.

Figure 1A indicates that among all groups in Comoros, the number of malaria cases, deaths, and DALYs declined markedly from 2010 to 2014 and remained at low levels between 2015 and 2017. From 2018 to 2019, the number of malaria cases increased again, followed by a decline in 2020. Malaria deaths and DALYs increased in 2018 and declined in 2019, and all indicators rebounded in 2021. Specifically, in the overall population, the number of malaria cases fell from 126,651 (95% UI: 102,670–157,081) in 2010 to 12,384 (95% UI: 10,210–14,942) in 2021, representing a −90.22% reduction; malaria deaths decreased from 540 (95% UI: 288–843) in 2010 to 30 (95% UI: 13–50) in 2021, indicating a −94.44% decline; and DALYs dropped from 34,724 (95% UI: 19,541–52,520) in 2010 to 1778 (95% UI: 886–2878) in 2021, which is a −94.88% reduction. Although the changing trends of these indicators were similar between males and females, the overall reductions were more pronounced among males (Table 1). Figure 1B indicates that in sub-Saharan Africa, these indicators, across all groups, changed more gradually. From 2010 to 2014, the number of malaria cases decreased slightly and then rose, while the number of deaths and DALYs declined slightly from 2010 to 2017 before increasing. Both deaths and DALYs fell again in 2021. In the overall population, the number of malaria cases rose from 221,381,206 (95% UI: 185,049,437–269,945,919) in 2010 to 229,191,055 (95% UI: 181,685,394–293,064,373) in 2021, reflecting a +3.53% increase, with a more substantial rise among females. Conversely, malaria deaths declined by −7.44%, from 759,438 (95% UI: 406,996–1,232,590) in 2010 to 702,937 (95% UI: 262,494–1,397,708) in 2021, with a more marked reduction among males. DALYs decreased by −11.36%, from 58,907,957 (95% UI: 33,290,778–93,197,378) in 2010 to 52,215,321 (95% UI: 21,277,913–99,016,298) in 2021, with a more pronounced decrease among females (Table 1).

**Figure 2:**
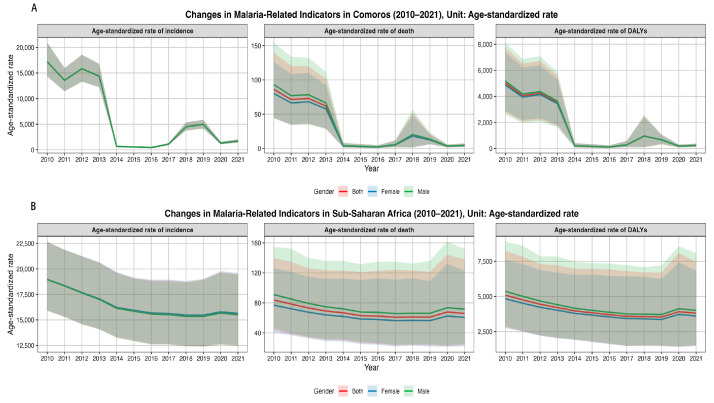
Temporal trends in the age-standardized rates (ASRs) of incidence (ASIR), mortality (ASMR), and DALYs (ASDR) in Comoros and sub-Saharan Africa from 2010 to 2021, stratified by total population, males, and females: (**A**) Comoros; (**B**) sub-Saharan Africa. DALYs, disability-adjusted life years.

Figure 2A demonstrates that the trends in ASIR, ASMR, and ASDR for malaria across all groups in Comoros mirrored the trends observed in their absolute values. For the entire population, the ASIR declined from 17,200.31 (95% UI: 14,327.51–20,935.67) per 100,000 in 2010 to 1687.94 (95% UI: 1395.05–2031.47) per 100,000 in 2021, yielding an EAPC of −18.70% (95% confidence interval [CI]: −33.77 to −0.20). The ASMR fell from 86.34 (95% UI: 44.15−139.7) per 100,000 in 2010 to 4.26 (95% UI: 1.91−7.24) per 100,000 in 2021, with an EAPC of −23.89% (95% CI: −36.58 to −8.66); and the ASDR fell from 5011.98 (95% UI: 2743 −7741.25) per 100,000 in 2010 to 234.27 (95%UI: 116.63−380.55) per 100,000 in 2021, with an EAPC of −24.49% (95% CI −36.88 to −9.66). Declines in ASMR and ASDR were greater in males, whereas ASIR trends showed no significant sex difference (Table 1). Notably, in 2021, all three indicators increased across all groups. Figure 2B shows that in sub-Saharan Africa, the ASRs of incidence, mortality, and DALYs among all groups gradually declined from 2010 to 2019, increased in 2020, and slightly decreased in 2021. For the entire population, ASIR decreased from 18,964.03 (95%UI: 15,909.77−22,663.51) per 100,000 in 2010 to 15,578.40 (95%UI: 12,473.58−19,528.01) per 100,000 in 2021, with an EAPC of –1.78% (95% CI: −2.35 to −1.21). ASMR fell from 83.85 (95%UI: 43.26−139.85) per 100,000 in 2010 to 65.90 (95%UI: 23.66−137.94) per 100,000 in 2021, with an EAPC of −2.13% (95% CI −3.29 to −0.95). ASDR declined from 5096.46 (95%UI: 2814.33−8247.95) per 100,000 in 2010 to 3810.98 (95%UI: 1517.53−7452.61) per 100,000 in 2021, with an EAPC of −2.67% (95% CI: −3.77 to −1.55). Apart from a slight sex difference in the ASIR trend, the trends in the other indicators did not differ significantly between sexes (Table 1).

### 3.2. Sex and Age Patterns

**Figure 3:**
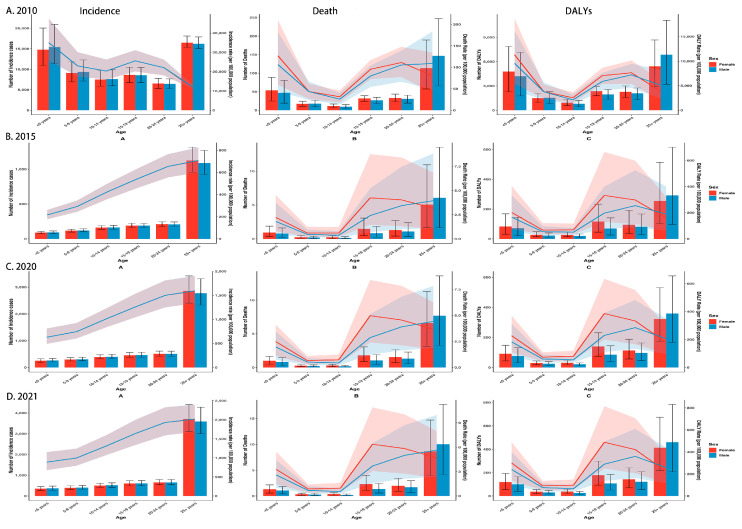
Trends in the number of malaria cases, deaths, DALYs and their crude rates across different age groups and sexes in Comoros: (**A**) 2010; (**B**) 2015; (**C**) 2020; (**D**) 2021. DALYs, disability-adjusted life years.

Figure 3 presents sex- and age-stratified trends in the number of malaria cases, deaths, DALYs, and their corresponding crude rates in Comoros for 2010, 2015, 2020, and 2021. In 2010, children under 5 exhibited the highest crude incidence rate (CIR) in both sexes. From 2015 onward, the peak CIR shifted to individuals aged 25+. Between 2010 and 2021, the CIR declined in every age stratum for both males and females, with the most significant reduction among children under 5. Nevertheless, from 2020 to 2021, the CIR rose in all age groups, and the rebound was most remarkable in the under-5 group. Crude mortality rate (CMR) patterns differed by sex. Throughout all study years, the highest CMR was recorded among males in adults aged 25+, whereas in females, the highest CMR occurred in the under-5 group in 2010, and from 2015 onward, the peak of CMR shifted to the 15–19 group. Between 2010 and 2021, CMR fell significantly in all age groups for both sexes, with the most significant decline among children aged 5 to 9. However, between 2020 and 2021, CMR increased again, most notably in males aged 5 to 9 and in females in the under-5 group. For crude DALY rate (CDR), males and females recorded the highest value in the under-5 group in 2010. By 2015, 2020, and 2021, the maximum had shifted to the 20–24 group for both sexes. Over the entire period, CDR declined sharply in every age category for both sexes, with the most substantial decrease in children aged 5 to 9. It increased again between 2020 and 2021 in all age groups, and the most considerable rise occurred in the under-5 group. Between 2010 and 2021, the number of malaria cases decreased in all age groups for both sexes, with the most significant reduction observed among children under 5. For both males and females, deaths decreased across all age groups, with the most pronounced decline in the 5–14 age group, while DALYs exhibited the most significant reduction in the 5–9 age group. However, between 2020 and 2021, both cases and DALYs increased for males and females, whereas deaths remained largely stable in most age groups (Tables S1 and S2).

**Figure 4:**
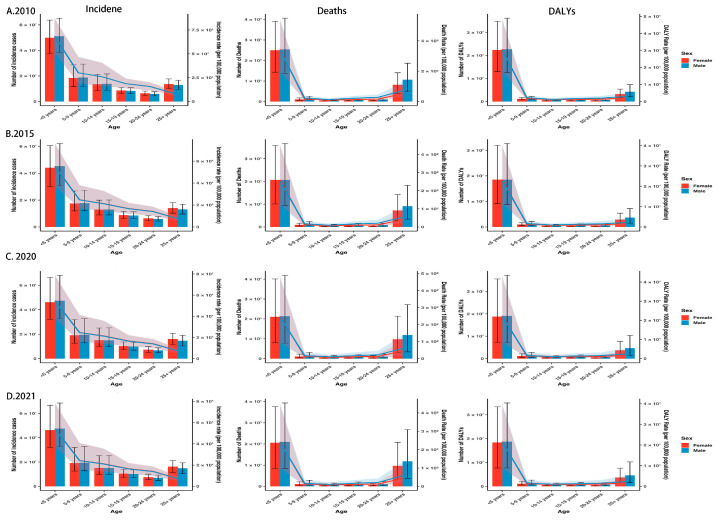
Trends in the number of malaria cases, deaths, and DALYs and their crude rates among different age groups and sexes in sub-Saharan Africa: (**A**) 2010; (**B**) 2015; (**C**)2020; (**D**) 2021. DALYs, disability-adjusted life years.

Figure 4 illustrates the trends in these indicators across different groups and age strata in sub-Saharan Africa in 2010, 2015, 2020, and 2021. Children under 5 consistently exhibited the highest CIR, CMR, and CDR in both sexes across all years. Among both males and females, all indicators declined over time across every age group, with the most significant reduction observed in the under-5 group. The pace of decline slowed between 2020 and 2021 for both sexes. Among males, the fastest decrease in CIR was observed in the 5–9 group, whereas the most rapid declines in CMR and CDR occurred in adolescents aged 15–19. Among females, CIR decreased most rapidly in the 5–9 group and among adults aged 25+, while CMR and CDR fell most sharply in the 5–9 group. From 2010 to 2021, case numbers declined only in the under-5 group and increased in all other age groups. In males, deaths and DALYs decreased in the under-5 and 5–9 groups but increased in other age groups. In females, deaths and DALYs decreased solely in the under-5 group, with increases in every other age stratum. Between 2020 and 2021, the number of malaria cases rose slightly in all age groups, whereas deaths and DALYs showed slight declines in most groups (Tables S1 and S2).

### 3.3. Decomposition Analysis

**Figure 5:**
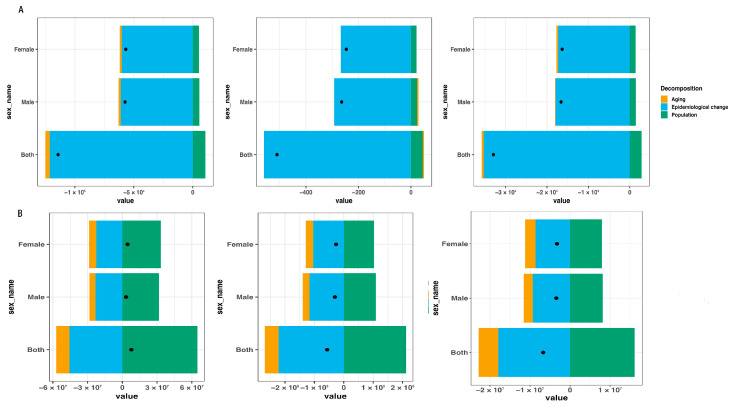
Changes in the number of malaria cases, deaths, and DALYs attributable to ageing, population growth, and epidemiological changes in Comoros and sub-Saharan Africa from 2010 to 2021, stratified by total population, males and females: (**A**) Comoros; (**B**) sub-Saharan Africa. DALYs, disability-adjusted life years.

The decomposition analysis results indicated that population growth contributed to an increase in the number of malaria cases, deaths, and DALYs, whereas epidemiological changes reduced these indicators. In Comoros, population growth contributed +9.36%, +8.82%, and +8.65% to the increase in the number of malaria cases, deaths, and DALYs, respectively. Conversely, epidemiological changes contributed −106.26%, −109.69%, and −107.04% to reductions in these indicators, respectively. Ageing contributed −3.11% and −1.61% to the decrease in the number of malaria cases and DALYs, respectively. Notably, among males, ageing contributed +1.79% to the increase in malaria deaths, whereas among females, it contributed −0.06% to the decrease in malaria deaths. In sub-Saharan Africa, population growth contributed +831.29%, +375.20%, and +241.14% to the increase in the number of malaria cases, deaths, and DALYs, respectively, while ageing contributed −146.56%, −83.43%, and −73.10% to the reductions in these indicators. Similarly, epidemiological changes contributed −584.72%, −391.78%, and −268.03% to the decreases in these indicators (Figure 5 and Table S3).

### 3.4. Risk Factors

**Figure 6:**
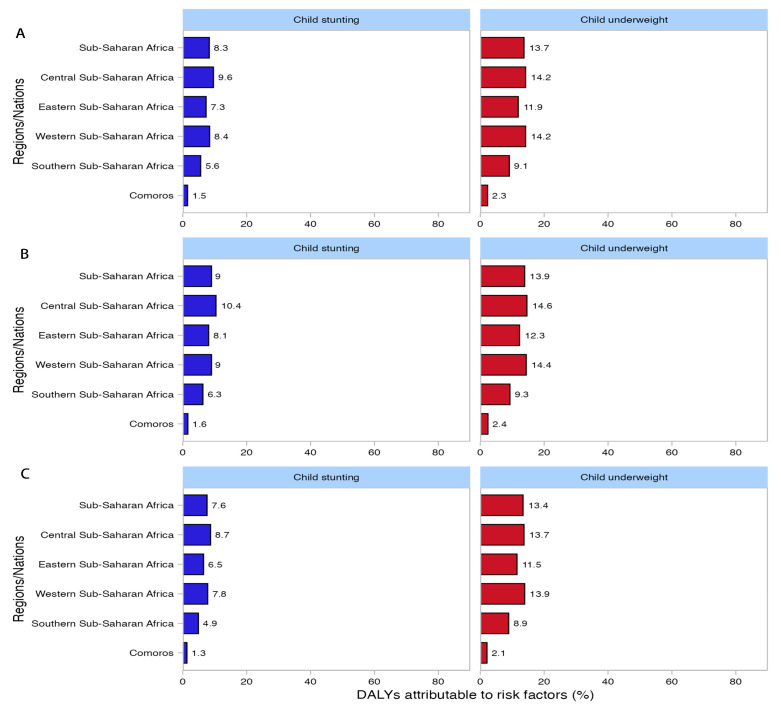
Percentage contribution of risk factors to the all-age DALYs of malaria in 2021 for the total population, males and females in Comoros, sub-Saharan Africa, and its subregions (Central, Eastern, Western, and Southern sub-Saharan Africa): (**A**) total population; (**B**) males; (**C**) females. DALYs, disability-adjusted life years, Blue, Child stuning, Red, Child underweight.

Figure 6 illustrates the contribution of two risk factors, namely, child underweight and child stunting, to all-age DALYs due to malaria among all groups in Comoros and sub-Saharan Africa and its subregions (Central, Eastern, Western, and Southern) in 2021. In the overall population of Comoros, child underweight (2.3%) and child stunting (1.5%) were the primary contributors to the malaria burden. In contrast, in sub-Saharan Africa and its subregions, these two risk factors were also the major contributors. Specifically, in the overall population of sub-Saharan Africa, child underweight and child stunting contributed 13.7% and 8.3% to the malaria burden, respectively. Furthermore, the contributions of these risk factors among males and females in these regions were consistent with the findings for the overall population, with child underweight consistently contributing more than child stunting (Figure 6B,C). Similarly, among the various population groups in the 46 countries of sub-Saharan Africa, the contribution of child underweight also surpassed that of child stunting (Figure S1).

## 4. Discussion

Over the past three decades, substantial progress has been made in the control and elimination of malaria worldwide, primarily due to the widespread adoption of artemisinin-based combination therapies (ACTs), improved preventive measures, and increased investment in malaria-control programs [16]. Nevertheless, the pace of progress has slowed appreciably since 2015 [17], and the COVID-19 pandemic has further disrupted malaria-control efforts, reversing earlier gains, particularly in the WHO African Region [3]. In 2023, this region recorded approximately 246 million malaria cases and 569,000 deaths, representing increases of +17.1% and +3.5%, respectively, relative to 2015 [3]. These figures indicate that the global malaria response is no longer on track to meet the GTS milestone of a ≥75% reduction in incidence and mortality rates by 2025 [8]. Our analysis further demonstrates that between 2010 and 2021, progress in sub-Saharan Africa was limited: malaria cases rose by +3.53%, whereas deaths and DALYs fell only by −7.44% and −11.36%, respectively. ASIR, ASMR, and ASDR declined slightly, with EAPCs of −1.78% (95% CI: −2.35 to −1.21), −2.13% (95% CI: −3.29 to −0.95), and −2.67% (95% CI: −3.77 to −1.55), respectively. Women experienced a proportionally more significant rise in cases, plausibly reflecting the heightened susceptibility of pregnant women [18]. Multiple factors contributed to this uptick in malaria cases. First, rapid population growth in malaria-endemic areas has expanded the size of the susceptible population, particularly in high-risk groups such as children [3,18]. Second, international assistance slowed: the annual growth rate of malaria funding fell from 28.9% (2000–2010) to 3.5% (2011–2019), while domestic spending rose by only 4.3% per year from 2000 to 2017 [16]. As sub-Saharan Africa is the principal recipient of this aid, the region experienced a corresponding reduction in funding support for malaria. These funding constraints led to shortages of ACTs, rapid diagnostic tests (RDTs), long-lasting insecticidal nets (LLINs), and indoor residual-spraying (IRS) supplies. In malaria-endemic regions, population growth enlarges the pool of susceptible individuals. At the same time, the persistent global funding gap for malaria control, which grew to USD 3.8 billion in 2021 [19], has led to inadequate coverage of key interventions such as LLINs and IRS, thereby undermining control efforts and driving up case numbers. Shortages of medical resources such as antimalarial drugs and diagnostic reagents impede the timely and effective treatment of malaria patients and extend the period during which infected individuals act as reservoirs of infection. This, in turn, facilitates further parasite transmission by mosquitoes to healthy hosts and exacerbates the risk of malaria spread. Third, *Plasmodium* falciparum strains with partial resistance to artemisinin and partner drugs emerged and spread [20]. Modeling denotes that widespread drug-resistant strains could generate an additional 78 million cases over the next five years, also posing a significant threat to ongoing control efforts. Fourth, plentiful rainfall and high temperatures provide ideal breeding conditions for female Anopheles mosquitoes [21], and growing resistance to pyrethroids and other insecticides in female Anopheles mosquitoes has eroded LLIN and IRS effectiveness [22]. Fifth, increasing *pfhrp2/3* gene deletions in malaria parasites have undermined the sensitivity of *HRP2*-based RDTs [23], thereby weakening the effectiveness of rapid diagnostic test (RDT)-based management strategies. Finally, persistent poverty fosters mosquito breeding sites and impedes access to adequate healthcare [24]. All these challenges have contributed to the expansion of the susceptible population and have undermined the coverage and effectiveness of key malaria control measures and treatment, thereby facilitating an increase in malaria cases. In addition, improved diagnostic capacity has also contributed to the apparent rise in reported cases. The COVID-19 pandemic compounded the malaria burden: in 2020, sub-Saharan Africa experienced a rebound in malaria cases, deaths, and DALYs, mirroring previous studies [3,25]. The primary drivers of this resurgence were disruptions in antimalarial supply chains and overstretched health systems that hindered preventive and treatment services for malaria [5].

By comparison, Comoros achieved substantial gains. The number of malaria cases, deaths, and DALYs decreased by more than −90%, and the EAPCs for the age-standardized incidence, mortality, and DALY rates each indicated declines exceeding −18% during the same period. Specifically, from 2010 to 2014, malaria cases, deaths, DALYs, and their ASRs fell sharply and remained low until 2017 before notably resurging in 2018. In 2020, malaria cases and ASIR declined, whereas malaria deaths, DALYs, and their age-standardized rates decreased in 2019; all indicators rose again in 2021. The WHO 2024 Malaria Report recorded continued increases, reaching 20,675 cases in 2022 and 21,049 in 2023 [3]. Malaria in Comoros is almost exclusively attributable to *P. falciparum*, with *P. vivax*, mixed infections, and other cases comprising only a tiny fraction. Moreover, after 2013, all reported malaria cases were solely due to *P. falciparum* infection (Table S4). The cornerstone of Comoros’ success in malaria control was the implementation of an integrated strategy centered on mass drug administration (MDA) combined with vector control, case management, and intermittent preventive treatment in pregnancy (IPTp). MDA is defined as several rounds of antimalarial drugs delivered to ≥80% of targeted individuals [26], aiming to reduce incidence and mortality rapidly and prevent resurgence. This comprehensive MDA strategy was implemented in Mohéli (2007), Anjouan (2012), and Grande Comore (2013) [27,28,29]. During this period, China’s antimalaria team coordinated multi-sectoral efforts to fight malaria, provided long-term training for primary healthcare staff and community volunteers, and supported mobile testing stations for malaria [30]. There were continuous declines in both malaria incidence and mortality after the intervention concluded [27,28,29]. Our findings also indicate that this comprehensive approach produced a sustained (around 3-year) decline in malaria epidemiological and disease burden indicators, far longer than the 1–3-month effect typically observed with MDA alone [1]. The 2018 reversal can be attributed to three reasons. First, the at-risk population grew by 8.1% between 2014 and 2018 [31]. Second, local authorities relaxed surveillance and control measures [31], imported cases rose (Table S4) [3], community engagement declined [32], and attrition of malaria control personnel weakened the already fragile medical system. Third, external funding contracted sharply; Global Fund disbursements in 2018 were 107% lower than in 2014 [31,33]. Consequently, LLIN distribution fell by 10.3% relative to 2017, and coverage of RDTs and IRS was unclear in 2018. Evidence indicates that once low transmission is achieved, sustained investment in establishing a malaria surveillance system and in training personnel is essential to ending malaria epidemics and preventing malaria resurgence [34]. In Comoros, during the maintenance phase (2015–2017), funding was only 37.6% of the 2010–2014 control-phase budget [33,35], thus disrupting the provision of malaria services to some extent and impeding progress toward malaria elimination. As a result, malaria rebounded in 2018. The authorities launched a new round of MDA on Grande Comore (Ngazidja) in 2019, but other interventions were incomplete; modeled ITN coverage was only 48.1%, and data on IRS, RDTs, and IPTp were lacking [5]. Thus, the protective effect of this round of MDA persisted for approximately one year. Moreover, the COVID-19 pandemic further disrupted antimalarial supply chains and interrupted malaria-related services. At the same time, weakened malaria surveillance led to increased imported cases post-pandemic (Table S4). It is noteworthy that in 2023, the population at risk in Comoros expanded further, yet coverage of key interventions such as vector control, chemoprevention for pregnant women, and MDA remained insufficient [3]. Consequently, malaria incidence continued to rise in 2023. MDA can swiftly reduce malaria burden in emergency settings and areas of moderate-to-high transmission, while also suppressing *Plasmodium falciparum* in very-low-to-low-transmission contexts and curbing *P. vivax* transmission [1]. Effective implementation hinges on four inter-locking stages: (i) a national-level design phase that secures high-level political commitment, establishes a coordinating body, characterizes the local epidemiological landscape, delineates target populations and areas, selects and procures antimalarial drugs, defines delivery strategies, and allocates budgetary resources; (ii) a provincial- and district-level planning phase in which teams draft detailed micro-plans encompassing population mapping, logistics and site selection, workforce training, pharmacovigilance, waste management, and robust community-engagement and risk-communication campaigns; (iii) an implementation phase that deploys pre-positioned drug kits through door-to-door or fixed-site approaches, underpinned by multi-tier supervision, real-time data capture for coverage and adverse events, and rapid coordination for problem solving; and (iv) a continuous monitoring-and-evaluation phase that quantifies coverage and drug consumption, conducts post-MDA surveys, maintains pharmacovigilance and resistance surveillance, and assesses impact using routine and parasitological data, culminating in comprehensive reporting of outcomes, challenges, lessons learned, and costs [36]. In Comoros, an area of intense transmission, MDA can substantially mitigate malaria burden provided it is embedded within an integrated control strategy that coordinates multiple rounds of drug distribution, sustains vector-control interventions, offers chemoprevention to high-risk groups, like pregnant women, strengthens health-worker capacity, fosters intensive community engagement, and maintains resilient surveillance-and-response systems. Long-term effectiveness and sustainability further depend on multi-sectoral collaboration that guarantees stable financing, an uninterrupted supply of drugs and vector-control commodities, sustained governmental support, broad community participation, and adaptive, data-driven program management. Consequently, future large-scale roll-outs should position MDA within an integrated control framework. They should continuously reinforce the implementation of support mechanisms that preserve their efficacy while facilitating a gradual transition to routine, sustainable malaria control.

The age-specific profile of malaria incidence has shifted in Comoros. Historically, children < 5 years bore the highest crude incidence rate (CIR), but adults ≥25 years now predominate, likely owing to greater outdoor exposure among adults, which increases their exposure to mosquitoes and the risk of infection, and to the improved protection of young children. During the COVID-19 period, however, cases and CIR rose most sharply in children < 5 years. In sub-Saharan Africa, by contrast, children < 5 years remain the principal risk group. Decomposition analysis revealed that between 2010 and 2021, population growth increased malaria cases, deaths, and DALYs in Comoros. This increase may be attributed to an increase in the number of susceptible newborns who have lost maternal immunity and have not yet developed specific immunity to malaria infection [37] and to the expansion of the population at risk in Comoros [3]. Meanwhile, effective malaria control measures remain insufficiently covered, thus increasing the burden of malaria. Epidemiological changes, by contrast, drive substantial declines attributable to improved malaria service capacity, organized MDA, comprehensive vector control, IPTp, and the establishment of local antimalaria teams, the improvement of their capacity, and so on. Ageing contributed to declines in both malaria cases and DALYs. This finding may be explained by the fact that in malaria-endemic areas, increasing age is associated with lower rates of parasitic infection and malaria incidence due to immune system maturation and the development of acquired immunity [37]. However, among males, ageing was associated with an increase in malaria deaths, whereas among females, ageing correlated with a decrease in malaria deaths. This difference can be attributed to the increase in the malaria CMR among males aged ≥25 years, whereas in females of the same age group, the malaria CMR exhibited a declining trend. For sub-Saharan Africa, decomposition analysis similarly indicates that population growth was the primary driver of rising malaria cases, deaths, and DALYs, whereas epidemiological shifts and ageing contributed to their reduction. Risk-factor analysis identified undernutrition, specifically child underweight and child stunting, as major determinants of malaria burden. Protecting children through nutritional interventions, vector control, and vaccination is therefore critical. Although the RTS, S/AS01 is projected to avert tens of thousands of deaths annually, its rollout faces numerous hurdles [38]. Consequently, conventional vector-control measures remain essential until the vaccine is fully deployed.

## 5. Limitations

There are several limitations in the study. First, although the GBD database is broadly trusted for its quality and reliability, potential flaws in its data-collection processes may limit the extent to which our results can be generalized. Second, some reported estimates have wide confidence intervals. This wide uncertainty likely arises from limited data sources, uneven reporting quality, and the simplifying assumptions within the models, which can also affect our results. Third, the relatively short observation period during the COVID-19 pandemic for changes in malaria epidemiological and disease burden indicators makes it challenging to assess the pandemic’s long-term impact on these trends in Comoros and sub-Saharan Africa. Fourth, directly comparing Comoros’ data with those of sub-Saharan Africa, without adjusting for differences in population size, health-system capacity, or reporting standards, may introduce bias and thus undermine the robustness of our trend analyses.

## 6. Conclusions

Existing interventions in Comoros can achieve malaria control. However, the primary causes of the malaria rebound in Comoros include the increased at-risk population, reduced funding for malaria control, shortages of antimalarial commodities, a fragile local healthcare system, an inadequate surveillance system, the loss of specialized malaria personnel, insufficient public engagement, and challenges similar to those encountered in sub-Saharan Africa. All of these factors have been exacerbated by the COVID-19 pandemic and remain unresolved. The incidence of malaria has also shifted: adults ≥25 years now account for most infections in Comoros. To curb the rebound and advance toward malaria elimination, Comorian authorities should address these challenges.

## Figures and Tables

**Table 1:** Temporal trends in the number of malaria cases, deaths, and DALYs and their age-standardized rates (ASRs) in Comoros and sub-Saharan Africa from 2010 to 2021, stratified by total population, males, and females.

Measure	Location	Sex	2010	2021	2010–2021	2010	2021	2010–2021
			Number (95%UI)	Number (95%UI)	RC (%)	ASR (per 10^5^, 95%UI)	ASR (per 10^5^, 95%UI)	EAPC (%, 95%CI)
Incidence	Comoros	Both	126,651 (102,670 to 157,081)	12,384 (10,210 to 14,942)	−90.22	17,200.31 (14,327.51 to 20,935.67)	1687.94 (1395.05 to 2031.47)	−18.70 (−33.77 to −0.20)
		Male	63,625 (51,485 to 79,017)	6163 (5080 to 7439)	−90.31	17,200.31 (14,327.51 to 20,935.67)	1687.94 (1395.05 to 2031.47)	−18.70 (−33.77 to −0.20)
		Female	63,026 (51,184 to 78,064)	6221 (5131 to 7503)	−90.13	17,200.31 (14,327.51 to 20,935.67)	1687.94 (1395.05 to 2031.47)	−18.70 (−33.77 to −0.20)
	Sub-Saharan Africa	Both	221,381,206 (185,049,437 to 269,945,919)	229,191,055 (181,685,394 to 293,064,373)	3.53	18,964.03 (15,909.77 to 22,663.51)	15,578.4 (12,473.58 to 19,528.01)	−1.78 (−2.35 to −1.21)
		Male	110,937,613 (92,711,851 to 135,452,396)	114,158,022 (90,433,854 to 146,696,541)	2.90	18,953.67 (15,903.46 to 22,649.87)	15,512.62 (12,426.43 to 19,437.26)	−1.82 (−2.4 to −1.24)
		Female	110,443,593 (92,337,586 to 134,516,767)	115,033,033 (91,251,540 to 146,367,832)	4.16	18975.46 (15917.4 to 22678.50)	15,643.46 (12,520.36 to 19,617.03)	−1.75 (−2.31 to −1.17)
Death	Comoros	Both	540 (288 to 843)	30 (13 to 50)	−94.44	86.34 (44.15 to 139.70)	4.26 (1.91 to 7.24)	−23.89 (−36.58 to −8.66)
		Male	279 (137 to 442)	15 (6 to 25)	−94.62	93.21 (43.88 to 154.54)	4.41 (1.87 to 7.67)	−24.18 (−36.89 to −8.91)
		Female	261 (150 to 399)	15 (7 to 25)	−94.25	80.24 (44.74 to 126.22)	4.15 (1.95 to 6.94)	−23.56 (−36.22 to −8.38)
	Sub-Saharan Africa	Both	759,438 (406,996 to 1,232,590)	702,937 (262,494 to 1,397,708)	−7.44	83.85 (43.26 to 139.85)	65.9 (23.66 to 137.94)	−2.13 (−3.29 to −0.95)
		Male	400,693 (208,524 to 668,953)	370,507 (135,482 to 752,024)	−7.53	91.06 (45.95 to 154.46)	71.7 (25.28 to 151.95)	−2.11 (−3.33 to −0.89)
		Female	358,745 (198,155 to 573,656)	332,430 (126,249 to 649,233)	−7.34	77.01 (40.35 to 126.36)	60.63 (22.11 to 122.95)	−2.11 (−3.22 to −0.98)
DALYs	Comoros	Both	34,724 (19,541 to 52,520)	1778 (886 to 2878)	−94.88	5011.98 (2743 to 7741.25)	234.27 (116.63 to 380.55)	−24.49 (−36.88 to −9.66)
		Male	17,458 (9118 to 26,594)	848 (397 to 1398)	−95.14	5175.58 (2613.65 to 8124.69)	228.46 (106.15 to 379.61)	−24.89 (−37.35 to −9.95)
		Female	17,266 (10,389 to 25,920)	930 (484 to 1488)	−94.61	4872.26 (2875.94 to 7377.94)	241.11 (125.55 to 386.05)	−24.08 (−36.41 to −9.36)
	Sub-Saharan Africa	Both	58,907,957 (33,290,778 to 93,197,378)	52,215,321 (21,277,913 to 99,016,298)	−11.36	5096.46 (2814.33 to 8247.95)	3810.98 (1517.53 to 7452.61)	−2.67 (−3.77 to −1.55)
		Male	30,526,557 (16,596,104 to 49,497,914)	27,101,669 (10,802,601 to 52,299,987)	−11.22	5369.34 (2892.3 to 8899.31)	4020.39 (1542.91 to 8084.54)	−2.66 (−3.81 to −1.49)
		Female	28,381,400 (16,503,490 to 44,431,680)	25,113,652 (10,510,511 to 46,335,752)	−11.51	4836.1 (2753.76 to 7604.98)	3617.94 (1484.20 to 6852.74)	−2.66 (−3.72 to −1.59)

RC = (value_2021_ − value_2010_)/value_2010_ × 100%. ASR, age-standardized rate; DALYs, disability-adjusted life years; RC, relative change; EAPC, estimated annual percentage change.

## Data Availability

Publicly available datasets were analyzed in this study. These data can be found at https://ghdx.healthdata.org/ (accessed on 16 May 2024).

## Supplementary Materials

The following supporting information can be downloaded at https://www.mdpi.com/article/10.3390/tropicalmed10050138/s1: Table S1: Trends in the crude rates of malaria incidence, mortality, and DALYs across different age groups and sexes in Comoros and sub-Saharan Africa for the years 2010, 2015, 2020, and 2021; Table S2: Trends in the number of malaria incidence, mortality, and DALYs across different age groups and sexes in Comoros and sub-Saharan Africa for the years 2010, 2015, 2020, and 2021; Table S3: Changes in the number of malaria cases, deaths and DALYs caused by ageing, population growth, and epidemiological changes among the entire population, males and females, 2010–2021; Table S4. The distribution of different types of malaria data and imported malaria cases in Comoros from 2010 to 2021; Figure S1: Percentage contribution of risk factors to the all-age DALYs of malaria in 2021 for the total population, males and females in the 46 countries of sub-Saharan Africa: (A) total population; (B) males; (C) females.
