# Supplementary figures and images for: Changes in Malaria Patterns in Comoros from 2010 to 2021: A Comparative Study with Sub-Saharan Africa
Source: Trop Med Infect Dis. 2025 May 19;10(5):138. doi: 10.3390/tropicalmed10050138 (PMC12115432; doi:10.3390/tropicalmed10050138)

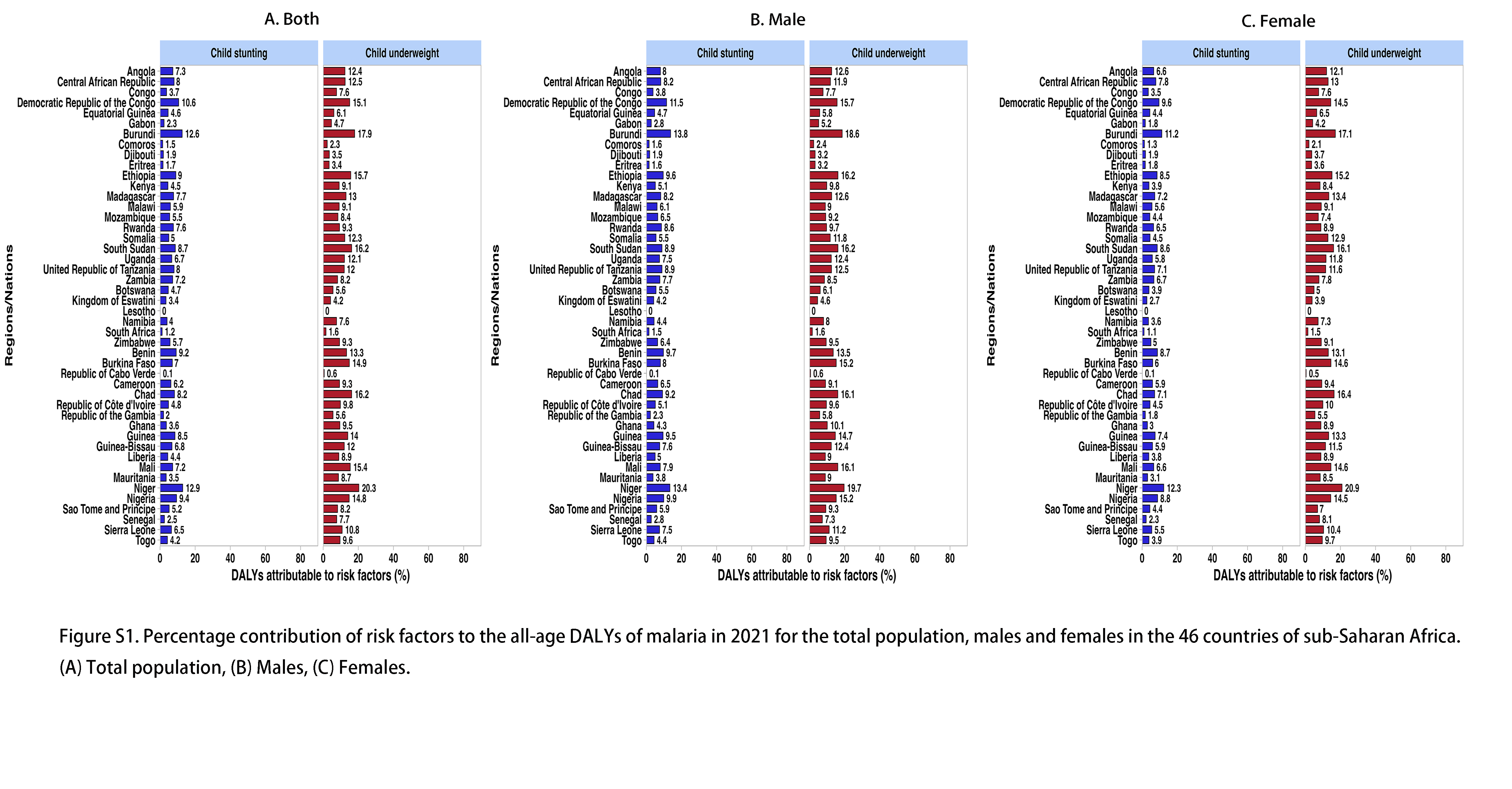

Supplement: Supplementary file 1 [file tropicalmed-10-00138-s001.zip › Figure S1.tif]
